# A *C. elegans* Screening Platform for the Rapid Assessment of Chemical Disruption of Germline Function

**DOI:** 10.1289/ehp.1206301

**Published:** 2013-04-19

**Authors:** Patrick Allard, Nicole C. Kleinstreuer, Thomas B. Knudsen, Monica P. Colaiácovo

**Affiliations:** 1 Department of Genetics, Harvard Medical School, Boston, Massachusetts, USA; 2 Department of Environmental Health Sciences, and; 3 Institute for Society and Genetics, University of California in Los Angeles, Los Angeles, California, USA; 4 National Center for Computational Toxicology, U.S. Environmental Protection Agency, Research Triangle Park, North Carolina, USA

**Keywords:** aneuploidy, *C. elegans*, chromosome segregation, germline, pesticides

## Abstract

Background: Despite the developmental impact of chromosome segregation errors, we lack the tools to assess environmental effects on the integrity of the germline in animals.

Objectives: We developed an assay in *Caenorhabditis elegans* that fluorescently marks aneuploid embryos after chemical exposure.

Methods: We qualified the predictive value of the assay against chemotherapeutic agents as well as environmental compounds from the ToxCast Phase I library by comparing results from the *C. elegans* assay with the comprehensive mammalian *in vivo* end point data from the ToxRef database.

Results: The assay was highly predictive of mammalian reproductive toxicities, with a 69% maximum balanced accuracy. We confirmed the effect of select compounds on germline integrity by monitoring germline apoptosis and meiotic progression.

Conclusions: This *C. elegans* assay provides a comprehensive strategy for assessing environmental effects on germline function.

Aneuploidy originates from chromosome segregation errors during the two highly regulated programs of cell division: mitosis and meiosis. Meiosis differs significantly from mitosis in that it reduces the number of chromosomes in half to produce haploid gametes: the egg and sperm. Human female meiosis, in particular, is inherently prone to errors, as evidenced by the high incidence and complexity of aneuploidies in stillbirths and spontaneous abortions (Hassold and Hunt 2001). While the etiology of aneuploidy is incompletely understood, evidence from mammalian studies suggests that exposure to diverse chemicals, including chemotherapeutic agents, alcohol, plastics, and pesticides, may be causative (Hales et al. 2005; Harkonen 2005; Hunt et al. 2003). However, despite the relevance of meiotic aneuploidies for reproductive health, we are currently unable to efficiently and comprehensively interrogate the multitude of chemicals in the environment for their effect on germline function and reproductive health.

Several programs at the National Institutes of Health (NIH), the U.S. Food and Drug Administration (FDA), and the U.S. Environmental Protection Agency (EPA) have identified a critical need in chemical risk assessment and initiated large-scale research programs (e.g., ToxCast, Tox21) using high throughput screening (HTS) assays for predictive toxicology (Dix et al. 2007; Kavlock et al. 2012; Krewski et al. 2010). In line with these efforts, we have developed an HTS platform for environmental toxicants based on germline dysfunction in the roundworm *Caenorhabditis elegans*. *C. elegans* offers significant advantages for this purpose: *a*) a high degree of conservation of key mammalian meiotic pathways, *b*) a well-studied model system of meiosis, and *c*) a vast array of available cytological, genetic, and biochemical tools (Colaiácovo 2006). Of particular interest was the use of *C. elegans* in a novel first-tier HTS strategy that would detect abnormal chromosome numbers. We chose to focus on environmental disruption of female meiosis because mammalian oogenesis encompasses events from early embryonic stages to adulthood and is therefore especially difficult to study. Here, we report the development of a platform that rapidly and comprehensively interrogates the landscape of environmental chemicals for potential effects on germline function, induction of aneuploidy, and prediction of mammalian reproductive deficits.

## Materials and Methods

C. elegans *genetics and growth conditions*. *C. elegans* strains were cultured as described by Brenner (1974) at 20°C on nematode growth medium (NGM) plates. The N2 Bristol strain was used as the wild-type strain. The following mutations and chromosome rearrangements were used in this study: LGIV, *col-121*(*nx3*), *him-8*(*e1489*); LGV, yIs34[*Pxol-1::GFP*, *rol-6*], bcIs39[*Plim-7::ced-1::GFP, lin-15*].

*Drug treatments and screening procedure*. All chemicals were purchased from Sigma-Aldrich (St. Louis, MO) and dissolved in dimethyl sulfoxide (DMSO; 0.1 M). Only chemicals that were dissolvable in DMSO at 0.1 M were considered for this screen. Final DMSO and chemical concentrations were 0.1% and 100 µM, respectively, except for mancozeb, dicofol, 2-(thiocyanomethylthio) benzothiazole (TCMTB), phosalone, chlorophene, endosulfan, and parathion-methyl, which were further diluted 10-fold to circumvent lethality. Chlorpyrifos-methyl was used at 1 µM for the same reason.

We synchronized an adult *C. elegans* population with sodium hypochloride treatment in order to generate age-matched embryos (Stiernagle 2006). The embryos were cultured on eight 10-cm NGM plates seeded with bacteria for 3 days at 20°C to generate a large pool of L4-stage worms that was resuspended in M9 buffer with bacteria. Live bacteria were used as described in numerous other chemical screens using *C. elegans* (e.g., Boyd et al. 2010a, 2010b), which, considering the screen’s relatively low false-positive and -negative rates, is not likely to be detrimental to this assay. After quantification under the microscope of the number of worms in population samples, 300 worms were dispensed in each well of a 24-well plate to which the chemicals were subsequently added. Each plate contained a negative control (0.1% DMSO) as well as a positive control (100 µM nocodazole). The worms were then incubated with shaking for either 24 hr or 65 hr at 25°C. After this incubation, the worms were transferred to 1.5-mL tubes, settled by gravity, and washed in M9 before being transferred to a slide and mounted with a coverslip for assessment of GFP^+^ embryos under an upright fluorescent microscope (Leica, Buffalo Grove, IL).

All statistical analyses performed after the *C. elegans* screen used the two-tailed Mann–Whitney *U* test with a 95% confidence interval (CI) unless specified otherwise.

*Predictivity analysis*. To assess the predictive value of the *C. elegans* screen against mammalian *in vivo* reproductive toxicity data, Toxicological Reference Database (ToxRefDB) (Martin et al. 2009) end points indicative of decreased female fertility were dichotomized with respect to their lowest effect level in a multigenerational study (MG-LEL). There were 47 compounds with multigenerational reproductive toxicity study data. Of those compounds, 20 had an MG-LEL of *≤* 500 mg/kg/day and were considered positive reproductive toxicants, and 27 had no MG-LEL in that range and were considered negatives. A subset of 7 compounds did not have associated ToxRefDB data; these were excluded from this portion of the analysis. The fold-change cutoff criteria for a positive hit in the *C. elegans* assay was iteratively increased from the lowest observed value in the assay to the highest, and sensitivity (true positive rate), specificity (true negative rate), and balanced accuracy (the average of sensitivity and specificity) were calculated for each cutoff value. A similar procedure based on iteratively increasing the cutoff value in the *C. elegans* assay at each time point and calculating the relative risk was followed for each individual multigenerational end point (with > 2 positive compounds). Statistical analysis was performed using R, version 2.13.0 (R Foundation, Vienna, Austria) [for code, see Supplemental Material, pp. 10–11, and Supplemental Source Code (http://dx.doi.org/10.1289/ehp.1206301)].

*Embryonic viability measurement*. Embryonic viability was performed three times for each exposure as described by Allard and Colaiácovo (2010). Briefly, the numbers of eggs laid and larvae hatched were recorded after a 24-hr exposure to DMSO and nocodazole.

*Apoptosis assay and germline nuclear analysis*. Quantitative analysis of germ cell apoptosis was performed using the *Plim-7::ced-1::GFP* strain as described by Saito et al. (2009). High-resolution images of germline defects were captured and processed as described by Allard and Colaiácovo (2010).

*Automated fluorescence reading*. A COPAS BIOSORT (Union Biometrica, Holliston, MA) was used for automated worm reading and sorting. Briefly, after a 24-hr exposure, the worms were washed at least three times in M9 buffer. The N2 wild-type strain was compared with *him8, Pxol-1::GFP* to ascertain the presence of GFP^+^ embryos and adjust reading settings accordingly. The reading parameters used were time-of-flight (ToF) for the *x*-axis and GFP peak height for the *y*-axis. The number of events per sample was 5,000, except for the *him-8* analysis where 1,000 events were read. A non-gated mixed population was used (mainly adults and embryos) from which only the objects of a size consistent with embryos were analyzed. The threshold to determine debris and GFP^–^ versus GFP^+^ embryos was set using a control population of untreated wild-type worms.

## Results

*Establishing a chemical screen for embryonic aneuploidy*. The strategy takes advantage of the rare proportion of male progeny (XO, < 0.2%) that naturally arises in wild-type hermaphroditic (XX) populations as a result of a meiotic segregation error of the X chromosome (Hodgkin et al. 1979). As disruption of meiosis very frequently leads to increased nondisjunction and aneuploidy, it correlates with a “high incidence of males” phenotype (Him), which is due to errors in X-chromosome segregation. This phenotype is also accompanied by an elevated embryonic lethality that follows from errors in autosomal chromosome segregation (Dernburg et al. 1998; Hodgkin et al. 1979). To easily detect male embryos *in utero* and circumvent embryonic lethality, a male-specific promoter (*xol-1*) is used to drive expression of GFP. This allows a quick identification of male embryos by the appearance of “green eggs” within the worm’s hermaphrodite uterus. The *Pxol-1::gfp* transcriptional reporter strain has been used in the context of a genetic screen, named the “Green eggs and Him” screen, which led to the isolation of an allele of the meiotic recombination factor, *msh-5* (Kelly et al. 2000; Nicoll et al. 1997).

**Figure 1:**
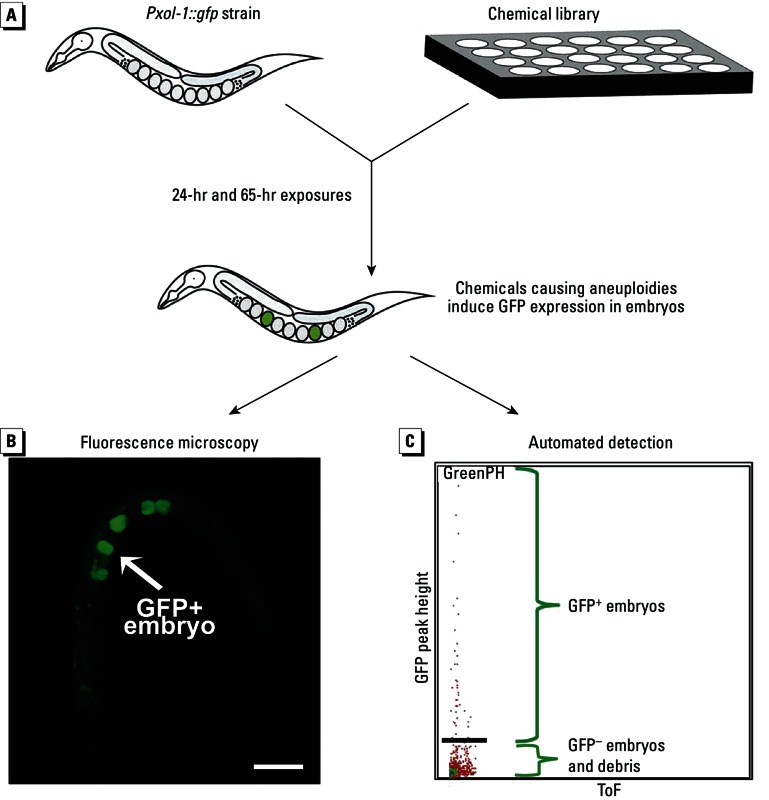
Design of the screening platform. (*A*) Worms from the aneuploidy-reporting *Pxol-1::GFP* strain are exposed to libraries of environmental compounds for either 24 hr or 65 hr. After exposure, the induction of aneuploidy can be visualized and quantified by fluorescence microscopy (*B*; bar = 100 µm) or automated detection and sorting of the worms (*C*). In *B*, several embryos expressing GFP (GFP+) can clearly be visualized. *C* shows automated reading of the embryos. A population of GFP+ embryos can be detected as distinct from GFP– embryos and debris, which appear below the black bar.

We developed a chemical strategy using the *Pxol-1::gfp* strain (Figure 1). Specifically, liquid cultures of the strain are exposed to chemicals of interest at 100 µM, a concentration commonly used in chemical screens in *C. elegans* (Boyd et al. 2010a, 2010b). The worm germline consists of nuclei simultaneously moving from the distal to the proximal end of the gonad and progressing through the meiotic stages in a synchronous manner. This establishes a spatial and temporal gradient of meiotic progression in *C. elegans*, with a well-characterized timing of events (Jaramillo-Lambert et al. 2007; Pazdernik and Schedl 2013). Consequently, we exposed the worms for durations of 24 hr and 65 hr in order to capture the effects of exposure at distinct stages of germline progression. Aneuploidies generated after a 24-hr test interval arise from the impairment of late meiotic (late pachytene and onward) and early embryonic processes, whereas the 65-hr interval captures aneuploidies originating from the disruption of any mitotic and meiotic events in the germline in addition to early embryonic stages. After exposure, the worms were readily observed under a fluorescence microscope. The number of GFP^+^ embryos were counted and normalized to the total number of embryos present to correct for decreased embryo production. We also established the automated detection and sorting of the GFP^+^ worms by using the COPAS BIOSORT (Union Biometrica) for sorting of viable worms and embryos. The use of a flow cytometry sorting system allowed us to scale up the numbers of chemicals being tested and the speed of screening, thus enabling high throughput capability (see below).

To discriminate between germline and embryonic chemically induced defects, we followed the fluorescence screen with two assays: *a*) a reporter-based germline apoptosis assay (Zhou et al. 2001), and *b*) DAPI-staining of the germline nuclei. These two complementary tests respectively measure induction of the meiotic DNA damage checkpoint (Gartner et al. 2008) and identify the nature of the germline nuclear defects responsible for apoptotic induction and the generation of aneuploidy.

**Figure 2:**
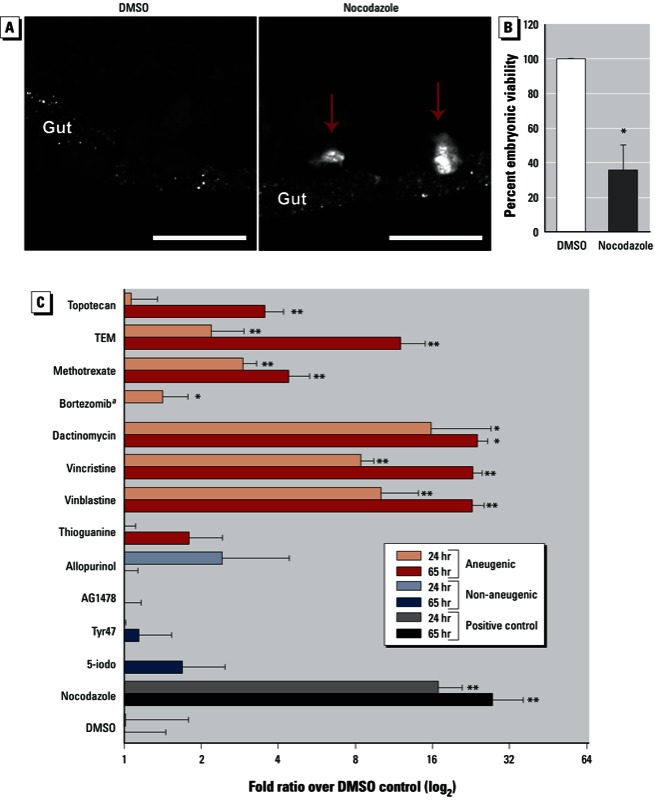
Chemical induction of aneuploidy in *C. elegans*. (*A*) *Pxol-1::GFP* worms were exposed to 100 µm nocodazole or 0.1% DMSO (control) for 24 hr. Two GFP+ embryos are visible within the nocodazole-treated worm’s uterus (arrows) adjacent to the autofluorescence emanating from the gut; bar = 50 µm. (*B*) Embryonic viability (mean percent ± SE) after either DMSO or nocodazole exposure. (*C*) Chemotherapeutic screen; the worms were exposed for 24 hr or 65 hr to 100 µM of each compound. The number of GFP+ embryos per worm was recorded, corrected for the average number of embryos found in each worm, and expressed as the log fold ratio over DMSO (mean percent ± SE; two-tailed Mann–Whitney *U* test, 95% CI, chosen over ANOVA with post-test correction to test for significant differences for each compound over DMSO because of high differences in sample variance). Each chemical exposure was performed six times. ^*a*^Lethal at 65 hr. *p ≤ 0.05, and **p ≤ 0.01, by two-tailed Mann–Whitney *U* test, 95% CI.

*Chemical induction of aneuploidy in* C. elegans *and determination of aneugenic potency*. Induction of aneuploidy in *C. elegans* has, to our knowledge, never been described in a chemical screening approach. To verify that *Pxol-1::gfp* reports chemical induction of aneuploidy, we tested exposure of these worms to the microtubule disruptor nocodazole. We expected nocodazole to promote chromosome segregation errors during the germline mitotic and late meiotic stages, as well as during early embryonic stages (Kitagawa and Rose 1999; Stear and Roth 2004). Thus, nocodazole should induce a high number of GFP^+^ embryos corresponding to increased X-chromosome missegregation. Indeed, worms exposed to 100 µM nocodazole for either 24 hr or 65 hr showed a statistically significant increase in the number of GFP^+^ embryos compared with DMSO alone (*p* = 0.002, Figure 2A,C). The increase in GFP^+^ embryos correlated with a 64% average decrease in embryonic viability, consistent with autosome missegregation (Figure 2B) (Hodgkin 2005).

For qualification of the assay, we next tested a set of reference compounds (chemotherapeutic agents) of well-defined aneugenicity. These chemicals have been used extensively in *in vitro* and *in vivo* tests to determine their aneuploidy-inducing potential in mammalian settings. The mode of action and published data describing their mammalian aneugenicity is presented in Supplemental Material, Table S1 (http://dx.doi.org/10.1289/ehp.1206301). We found that known aneugenic agents (bortezomib, dactinomycin, methotrexate, nocodazole, triethylenemelamine, topotecan, vinblastine sulfate, and vincristine) were statistically significant inducers of GFP^+^ embryos when compared with DMSO at both 24-hr and 65-hr time points. We observed that over the combined time points, seven of the eight aneugenic compounds were statistical hits, with microtubule drugs (nocodazole, vinblastine sulfate, and vincristine) showing the strongest levels of induction. Conversely, all four non-aneugenic compounds tested (5-iodotubercidin, AG1478, allopurinol, and Tyr47) were not different from controls (Figure 2C). The one false-negative, thioguanine, may have been missed because of the weak germline expression in *C. elegans* of hypoxanthine phosphoribosyltransferase 1 (HPRT1), an enzyme important for the metabolism and toxicity of thioguanine (Kohara and Shin-i 2013). Finally, bortezomib was toxic at the 65-hr time point but positive at 24 hr. All together, these results indicate that the *Pxol-1::*GFP reporter strain can be used in a chemical screening setting to accurately discriminate compound aneugenicity.

*Screening of environmental compounds with defined mammalian reproductive toxicity*. We hypothesized that aneugenic compounds disrupting germline chromosome segregation would likely cause reproductive impairment in mammals. Hence, aneugenic chemicals should be overrepresented among those whose exposure leads to decreased fertility and underrepresented among those showing no reproductive toxicity. To test this hypothesis, we mined the U.S. EPA’s ToxRefDB (http://www.epa.gov/ncct/toxrefdb/). This extensive resource compiles over 30 years of mammalian *in vivo* toxicity data on 474 chemicals, primarily pesticides and antimicrobials, and comprises several thousands of *in vivo* end points from chronic/subchronic carcinogenicity, prenatal developmental toxicity, and multigenerational reproductive toxicity studies (Knudsen et al. 2009; Martin et al. 2009). The majority of the chemicals in the ToxRefDB, and all of those in the present study, also have associated *in vitro* HTS data in the U.S. EPA’s ToxCast program across hundreds of human gene and protein targets (Kavlock et al. 2012).

**Figure 3:**
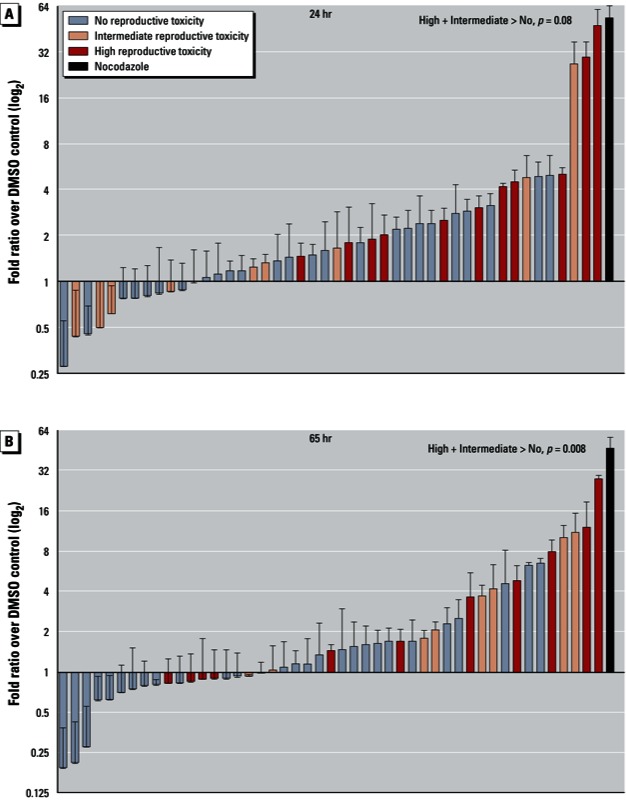
Screening of environmental chemicals. Worms were exposed for either 24 hr (*A*) or 65 hr (*B*) to each compound, at a concentration of 100 µM [except for mancozeb, dicofol, 2-(thiocyanomethylthio) benzothiazole (TCMTB), phosalone, chlorophene, endosulfan, parathion-methyl, which were further diluted 10-fold, and chlorpyrifos-methyl, which was used at 1 µM, to circumvent lethality]. The number of green embryos per worm was recorded and corrected for the average number of embryos found in each worm. The number was then expressed as the log fold ratio over DMSO. Chemicals are listed in order in Supplemental Material, Tables S2 and S3 (http://dx.doi.org/10.1289/ehp.1206301). The compounds were categorized according to their assessed mammalian reproductive toxicity [i.e., the number of mammalian end points for which they were positive: high reproductive toxicity (> 2 end points), intermediate reproductive toxicity (1 end point), and no reproductive toxicity (0 end points)]. At 65 hr, the mean value of fold-induction for the high and intermediate reproductive toxicity groups was significantly higher than the no–reproductive toxicity group (*p* = 0.008). Each chemical was tested three times.

We tested the utility of the meiotic screen by comparing results from a panel of 47 compounds with selected mammalian reproductive end points in ToxRefDB that were indicative of decreased female fertility. These *in vivo* end points included decreased implantation sites, litter size, early postnatal pup survival, overall reproductive success, reproductive performance, and fertility as well as ovarian morphology defects. The selected compounds were grouped into three categories according to the number of mammalian end points they were positive for: *a*) high reproductive toxicity (32 end points), *b*) intermediate reproductive toxicity (1 end point), and *c*) no reproductive toxicity (0 end points). The chemicals that were tested, their ranking by fold induction in the *C. elegans* assay, and their corresponding mammalian *in vivo* end point data are presented in Supplemental Material, Tables S2 and S3 (http://dx.doi.org/10.1289/ehp.1206301). As shown in Figure 3, at 65 hr, there is a statistically significant partitioning of all reproductive toxicants (high and intermediate) from compounds that are not reproductive toxicants (*p* = 0.008; two-tailed Mann–Whitney *U* test, 95% CI). The 24-hr exposure showed a trend toward significance (*p* = 0.08). These results indicate a clear enrichment of reproductive toxicants as positive hits from the screen, suggesting that chemical aneugenicity is a likely source of reproductive toxicity in mammals.

*Predicting mammalian reproductive impairment from the* C. elegans *screen*. We next assessed the predictive value of the *C. elegans* screen with respect to mammalian *in vivo* reproductive toxicity data, where compounds with an MG-LEL of *≤* 500 mg/kg/day were considered positive reproductive toxicants, and those with no MG-LEL in that range were considered negatives. This cutoff value approximates the reproductive test guideline testing limit of 1,000 mg/kg/day and accounts for the large uncertainty around dose measurements and standard conversions applied across many studies and over 30 years of toxicity testing. There was a subset comprising seven compounds that did not have associated ToxRefDB data; these seven compounds were excluded from this portion of the analysis. The data (log fold ratio over DMSO control) from both the 24-hr and 65-hr exposure intervals were used to predict mammalian reproductive toxicity. As shown in Supplemental Material, Figure S1A,B (http://dx.doi.org/10.1289/ehp.1206301), we calculated the maximum balanced accuracy, which corresponds to the average of sensitivity (ability to correctly identify true positives) and specificity (ability to correctly identify true negatives) and is, therefore, a representation of the predictive value of the screen. The balanced accuracy was 68% for the 24-hr exposure at a cutoff of 1.6, and 69% for the 65-hr exposure at a cutoff of 1.7. Interestingly, at these cutoff values the 24-hr exposure provided greater sensitivity (70%), whereas the 65-hr exposure provided greater specificity (78%). For the seven compounds without associated ToxRefDB guideline multigenerational study information, these cutoff criteria identified three positives at both time points (dimethomorph, niclosamide, and fenitrothion), two positives at 24 hr only (clorophene and HPTE), and one positive (methoxychlor) at 65 hr only. One compound, prochloraz, was negative at both time points (see Supplemental Material, Tables S2 and S3).

We then calculated the relative risk and associated confidence intervals for each mammalian end point indicative of decreased female fertility by iteratively varying the cutoff for a positive result in the *C. elegans* assay, from the lowest observed value to the highest, at each time point. The maximum relative risks for each end point, corresponding to a *C. elegans* assay cutoff between 1 and 2 (log fold ratio over DMSO control), are shown in Table 1. In certain cases, higher cutoff values produced larger relative risks, but at the expense of large numbers of false-negatives and extreme confidence intervals; therefore, we have reported the maximum relative risks corresponding to a cutoff range that optimized the predictive value of the assay. A cutoff of 1.71 at the 65-hr time point produced the highest relative risk score (9.69) for the multigenerational rat end point of ovary microscopic and gross pathologies and weight changes (termed MGR_Rat_Ovary). Although the 65-hr time point was most predictive overall for any multigenerational end point, the remainder of the end points had maximal relative risk scores ranging from 2.56 to 9.69 that were associated with cutoff values of 1.43 to 1.80 at the 24-hr time point. Supporting the strong bias toward predicting reproductive impairment, the screen is not predictive of other unrelated end points such as mammalian liver genotoxicity [see Supplemental Material, Figure S2 (http://dx.doi.org/10.1289/ehp.1206301)]. Together, the results show that the *C. elegans* screening strategy is predictive of mammalian reproductive toxicity, with a balanced accuracy approaching 70% and significantly increased relative risk values for reproductive impairment end points.

**Table 1:** Relative risk.

Reproductive toxicity end point	Relative risk (95% CI)	(+) Cutoff:*C. elegans* assay	Time point
MGR_Rat_Fertility	4.05 (0.35, 46.60)	1.80	24 hr
MGR_Rat_LitterSize	6.82 (0.82, 56.76)	1.64	24 hr
MGR_Rat_Ovary	9.69 (1.11, 84.53)	1.71	65 hr
MGR_rat_ReproductiveOutcome	8.08 (1.11, 58.93)	1.43	24 hr
MGR_rat_ReproductivePerformance	9.45 (1.19, 74.85)	1.80	24 hr
MGR_Rat_ViabilityPND4	2.56 (1.49, 2.39)	1.64	24 hr
mgLEL (any)	2.15 (1.75, 2.64)	1.69	65 hr
Abbreviations: MGR, multigenerational; PND4, postnatal day 4. For each reproductive toxicity end point, relative risk was calculated by iteratively increasing the cutoff value (log fold ratio over DMSO control) for a positive result in the*C. elegans* assay at each time point. Maximum relative risks and 95% CIs are shown for cutoff values within a range shown to maximize the predictive value of the assay.			

**Figure 4:**
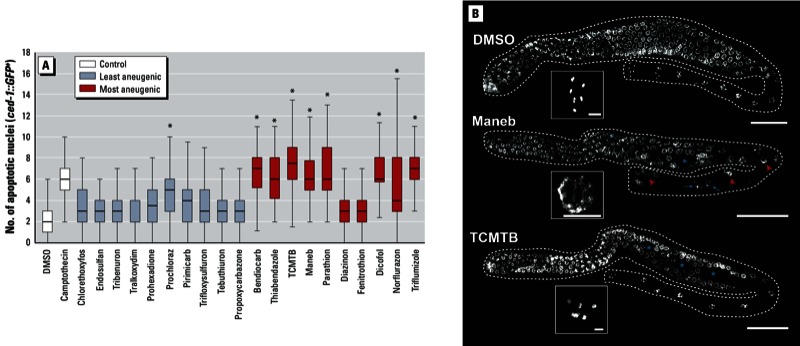
Functional validation of selected compounds. (*A*) Germline apoptosis assay quantification. After exposure to each of the 10 least aneugenic compounds in the *C. elegans* assay or to each of the 10 most aneugenic compounds, we quantified apoptotic levels through the use of the *Plim-7ced-1::gfp* reporter DMSO was the negative control, and the DNA damaging agent camptothecin the positive control. The lower and upper edges of the box plot represent the first and third quartiles, respectively, with the median represented as a line within the box; the whiskers extend to ± 1.5 times the interquartile range. (*B*) DAPI staining of germ­line nuclei revealed profound germ­line defects after exposure to Maneb and TCMTB (bars = 50 µm). Assembled germ­lines show areas of reduced nuclear density (asterisks), intermixed meiotic stages (red arrowheads), and unequally spaced diakinetic nuclei (white arrows). Insets show high magnification examples of late diakinetic nuclei (bars = 4 µm); note that nuclei with chromosomes in a pachytene stage-like organization are present intermixed with diakinetic nuclei in late prophase after maneb exposure. *p < 0.001 compared with DMSO by ANOVA followed by Dunn’s comparison. The numbers of apoptotic nuclei in the 10 least and most aneugenic compounds were highly significantly different from each other (*p* < 0.0001; two-tailed Mann–Whitney *U* test, 95% CI)

*Analysis of meiotic defects from selected compounds*. A critical aspect of the screen is the follow-up analysis, albeit not high throughput, of the chemical hits to discriminate between germline versus early embryonic defects as the source of aneuploidy. To this end, we first monitored the activation of the late pachytene meiotic checkpoint that leads to apoptotic clearing of nuclei carrying unrepaired DNA damage (Gartner et al. 2008). Here, we made use of a strain carrying the *Plim-7ced-1::gfp* transgene to specifically mark engulfed nuclei undergoing apoptosis (Zhou et al. 2001), and we compared the 10 most aneugenic compounds (based on fold change at 65 hr) with the 10 least aneugenic ones (Figure 4A). The difference in apoptotic levels between the two groups was extremely significant (*p* < 0.0001, by the two-tailed Mann–Whitney *U* test, 95% CI). The statistical comparisons with vehicle control (DMSO) are shown in Figure 4A. The baseline of apoptotic levels were slightly elevated compared with DMSO, and a stringent statistical cutoff of *p* = 0.0001 of comparison to DMSO was necessary to separate the most and least aneugenic chemicals. This test, however, clearly indicated a dramatic induction of germline apoptosis in many of the top hits from the screen.

Next, we confirmed the presence of meiotic defects in the groups with high levels of apoptosis. Specifically, we observed severe germline defects after exposure to the aneugenic compounds that also induced germline apoptosis [Figure 4B; see also Supplemental Material, Table S4 (http://dx.doi.org/10.1289/ehp.1206301)]. For example, worms exposed to the fungicide Maneb showed severe germline disorganization including gaps or areas with a reduced density of nuclei (Figure 4B), which may be due to either impaired meiotic progression or the degeneration of a fraction of nuclei, and a disorganization in the spatial/temporal gradient of meiotic stages (Figure 4B; evidence of intermixing of nuclei at different meiotic prophase stages). At the stage of diakinesis (end of prophase I), when fully cellularized oocytes are positioned in a single continuous row in wild-type worms, we also detected unevenly spaced nuclei, suggesting a defect in cytokinesis (white arrows). Interestingly, both gaps and intermixing of nuclei at different meiotic stages (red arrows) were also observed after exposure to the fungicide TCMTB. None of these defects was observed in the DMSO-exposed control worms or in animals exposed to other compounds (see Supplemental Material, Table S4). Together, these experiments strongly suggest a meiotic origin for the embryonic aneuploidy detected in the screen. This strategy therefore provides a fast and reliable tool to elucidate environmental influences on germline function and predict mammalian reproductive toxicity.

**Figure 5:**
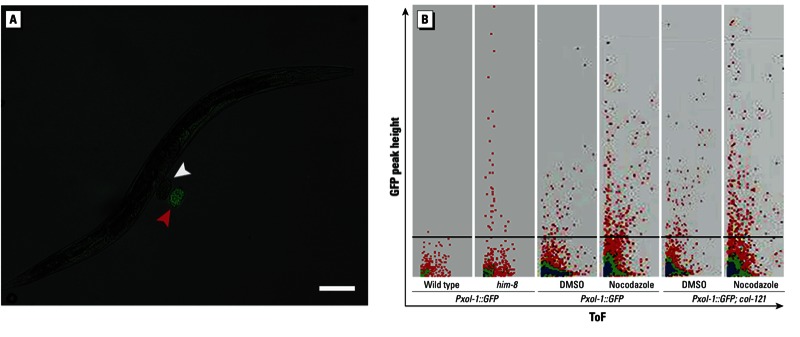
High throughput detection of genetic and chemical induction of aneuploidy. (*A*) Overlay of bright field and fluorescence images of a *Pxol-1::GFP*, *him-8* worm with two embryos; the GFP^+^ embryo is clearly distinguishable from the GFP^–^ embryo next to it (bar = 100 µm). (*B*) Automated detection of GFP^+^ embryos using the COPAS BIOSORT; the black line represents the GFP^+^ threshold as determined by *Pxol-1::GFP* without *him-8* (WT count). The GFP^+^ population of embryos is more abundant in nocodazole-treated worms compared with DMSO-treated worms in both the *Pxol-1::GFP* and the *Pxol-1::GFP; col-121* (sensitized) backgrounds.

*High throughput adaptation and chemical sensitization*. Finally, we propose a technology that can be readily applied in an HTS assay. We used an automated fluorescence-assisted sorter for large objects (COPAS BIOSORT, Union Biometrica) that can accurately read and sort whole animals as well as embryos from a suspension of worm culture (Boyd et al. 2010a, 2010b). To verify that the worm sorter can detect the presence of aneuploidy/GFP^+^ embryos, we first sorted two genetically distinct worm populations: *Pxol-1::gfp* and *Pxol-1::gfp; him-8*(*e1489*) worms. HIM-8 is a protein that associates with a region known as the pairing center on the X chromosome in *C. elegans* whose activity is essential for the proper segregation of the X chromosome during meiosis (Hodgkin et al. 1979). Thus, *him-8*(*e1489*) mutants produce a high number of male progeny (approximately 30%) due to increased X-chromosome missegregation that can be easily visualized in the context of the *Pxol-1::gfp; him-8*(*e1489*) strain (Figure 5A). Automated reading of the two populations easily identified a clear subset of GFP^+^ embryos that were present in much lower numbers in the *Pxol-1::gfp* worms alone, which allowed us to determine a threshold to discriminate between GFP^+^ and GFP^–^ embryos and any remnants of culture debris.

Next, we tested the sorting of worms exposed to nocodazole and compared it with worms exposed to DMSO (control worms). Automated reading readily identified two distinct groups based on fluorescence levels, with approximately 3-fold induction between nocodazole-treated and control worms (Figure 5B). We also chemically sensitized the *Pxol-1::gfp* strain by incorporating the mutant allele *nx3* of the cuticle collagen gene *col-121*, which was isolated in a screen for hypersensitivity to bisphenol A (Watanabe et al. 2005). In this background, a 2.7-fold increase in GFP^+^ embryos was observed in DMSO treated worms compared with *Pxol-1::gfp* alone, possibly because of increased sensitivity to the low aneugenic activity of DMSO (Goldstein and Magnano 1988). However, the number of GFP^+^ events captured was also dramatically improved: about 220% more for the same number of worms sorted. Thus, automated detection and sorting of the worms is a valuable option for the HTS screening of chemical aneuploidy.

## Discussion

Our results show the *C. elegans* assay is an efficient and reliable technology for the fast screening of chemicals altering germline function. We focused on environmental compounds as a mean to address a gap in our current ability to assess the potential hazards of thousands of untested chemicals. However, the assay described here is also applicable to other chemical screens, including drug safety assessment and small molecule assays for the analysis of germline pathways.

We estimate that the screening time with the COPAS BIOSORT (Union Biometrica) will consist of 65 hr of exposure followed by 45 min of reading for each 96-well plate. Because exposures can be performed simultaneously and each plate adds only an additional 45 min of reading time, a library of 1,000 compounds could be screened in triplicate in as few as 4 days. The running costs of the screen are extremely low given that the only reagents necessary for the screen are deep 96-well plates, buffer solution, and bacteria for food. By comparison, mammalian reproduction assays are much costlier and lengthier. A typical single-generation rodent reproduction test involves an 8- to 10-week exposure window starting around puberty and comprising ≥ 20 pregnancies for each dose group (U.S. EPA 1996). Furthermore, mammalian cell–based assays do not recapitulate efficiently all stages of meiosis and are not suitable for large-scale platforms. Thus, we are providing a unique whole organism first tier assay that examines the outcome of complex cellular and developmental processes with short running time, modest cost, and high accuracy.

The “Green eggs and Him” output is representative of overall levels of aneuploidy as evidenced by *a*) the correlation between GFP expression and the presence of germline defects as well as high levels of embryonic lethality, a phenotype expected from the missegregation of the autosomes and not just the X chromosome (Hodgkin 2005; Kelly et al. 2000); *b*) the fact that most genetic disruptions of the germline lead to missegregation of all chromosomes and not just the X chromosome (Dernburg et al. 1998; Hodgkin et al. 1979; Kelly et al. 2000); and *c*) that exposures to at least two hits from the screen, Maneb and TCMTB, show a high level of germline disruption, indicating that the GFP readout can indicate the disruption of germline processes. Nonetheless, although not performed in this study, a follow-up analysis of selected hits should include the measurement of embryonic lethality. This measurement requires significant time and cannot be embedded within an HTS assay. However, it will permit further validation of the hits as affecting other chromosomes besides the X chromosome.

An interesting feature of the screening results is that some of the strong aneuploidy-inducing hits [see Supplemental Material, Tables S2 and S3 (http://dx.doi.org/10.1289/ehp.1206301)] lack any described mammalian reproductive toxicity. Although we cannot explain the presence of all of the compounds near the top of the list, some of them have well-described mammalian germ cell aneugenicity [e.g., thiabendazole (ranked seventh at 65 hr; see Supplemental Material, Table S3)] (Mailhes et al. 1997; Schmid et al. 1999). The outcome of our screen, together with past aneugenic evidence, predicts a potential reproductive hazard of thiabendazole. Finally, some compounds are negative hits in the screen, implying that they produce less aneuploidy than DMSO alone. A possible explanation for this would be the undesirable direct inhibition of reporter expression. However, the number of chemicals exerting such an effect is low (2 of 47 chemicals at 24 hr and 65 hr) and is manageable in the context of a first pass screening strategy.

A difference in ADME (absorption, distribution, metabolism, and excretion) parameters, such as for thioguanine, will be a likely source of false-negatives in *C. elegans* compared with mammalian systems. Furthermore, a potential avenue for improving the screen could be the inclusion of dose–response curves for each compound. However, a distinct advantage to the present approach is the ability to screen with varying sensitivity or specificity depending on the application, be it risk assessment or the identification of mechanism of action. The cutoff criteria for a positive hit in the *C. elegans* assay may be optimized accordingly, where a value of 1.2 at the 24-hr exposure provides a sensitivity of 80%, and a value of 2.3 in the 65-hr exposure provides a specificity of 89%. Using this method removes many of the aforementioned false-negatives or false-positives, respectively. As shown in Table 1, higher relative risks for reproductive end points such as offspring viability and litter size corresponded to lower cutoff values at the 24-hr time point, and higher relative risk for end points such as fertility and ovarian pathology corresponded to higher cutoff values or the later 65-hr time point. The differing time points and cutoff values may provide information on varying events in the meiotic process, embryonic stages, or specific reproductive organs that may be targeted or impaired by different chemicals.

## Conclusion

With a low cost, high speed, and strong predictive value, this technology fulfills the requirement for first pass assessment of chemical hazard, and furthermore, it offers insight into germline disruption as a mechanism of reproductive toxicity.

## Supplemental Material

(2.2 MB) PDF
