# Supplementary material for: A C. elegans Screening Platform for the Rapid Assessment of Chemical Disruption of Germline Function
Source: Environ Health Perspect. 2013 Apr 19;121(6):717–24. doi: 10.1289/ehp.1206301 (PMC3672921; doi:10.1289/ehp.1206301)
Supplement: (2.2 MB) PDF [file ehp.1206301.s001.pdf]

Supplemental Material

A *C. elegans* Screening Platform for the Rapid Assessment of Chemical Disruption of Germline Function

Patrick Allard, Nicole C. Kleinstreuer, Thomas B. Knudsen, and Monica P. Colaiácovo

TABLE OF CONTENTS

Figure S1	Page 2
Figure S2	Page 3
Table S1	Page 4
Table S2 – 24 hour exposure	Page 5
Table S3 – 65 hour exposure	Page 7
Table S4	Page 9
R code for calculating relative risk values	Page 10
References	Page 12

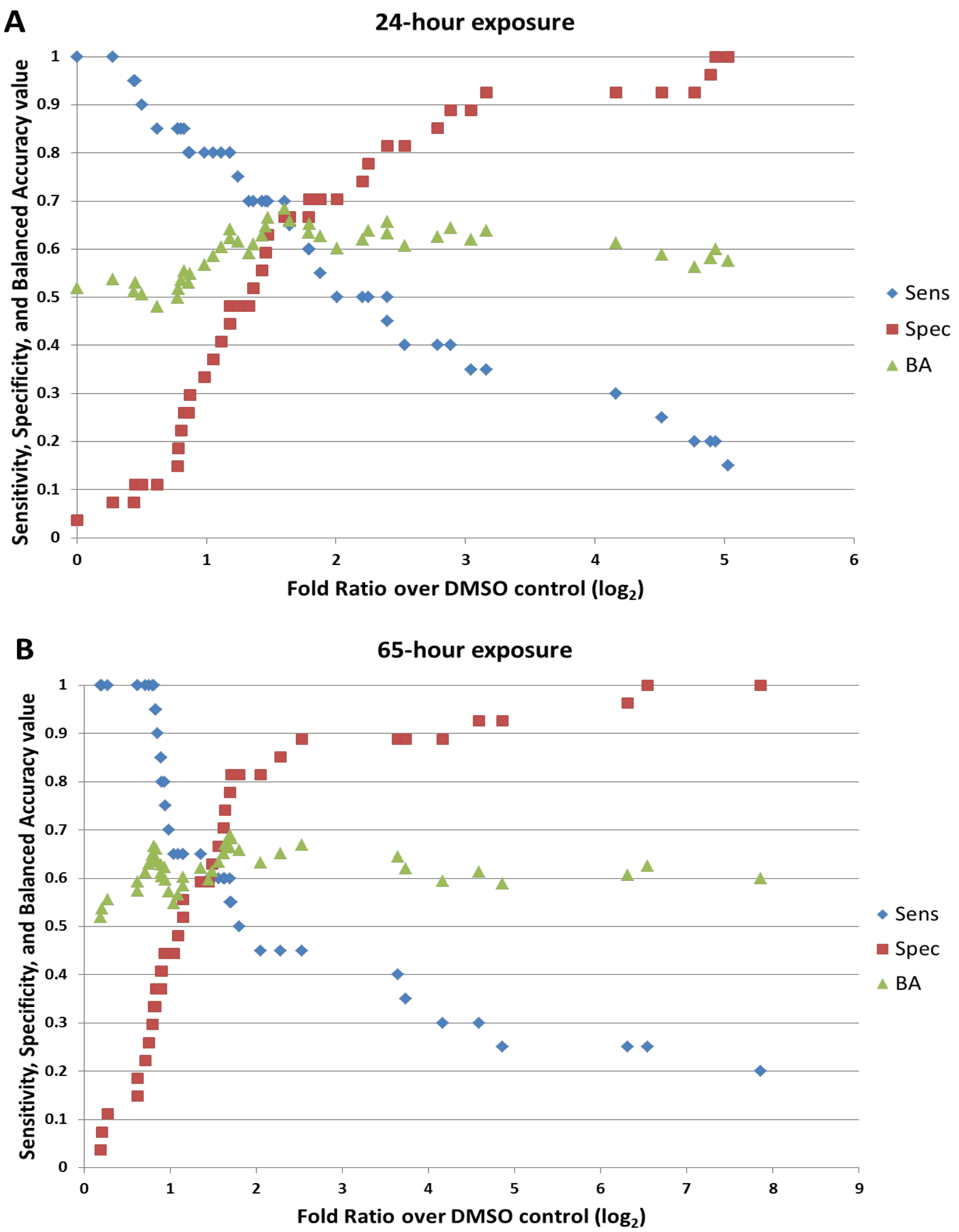

Supplemental Material, Figure S1. Predicting mammalian reproductive impairment from the *C. elegans* screen.

Predicting *in vivo* reproductive toxicants by iteratively varying the cutoff for a positive result in the *C. elegans* screen. Sensitivity, Specificity, and Balanced Accuracy are shown for (A) the 24-hour exposure and (B) the 65-hr exposure intervals.

Figure S2

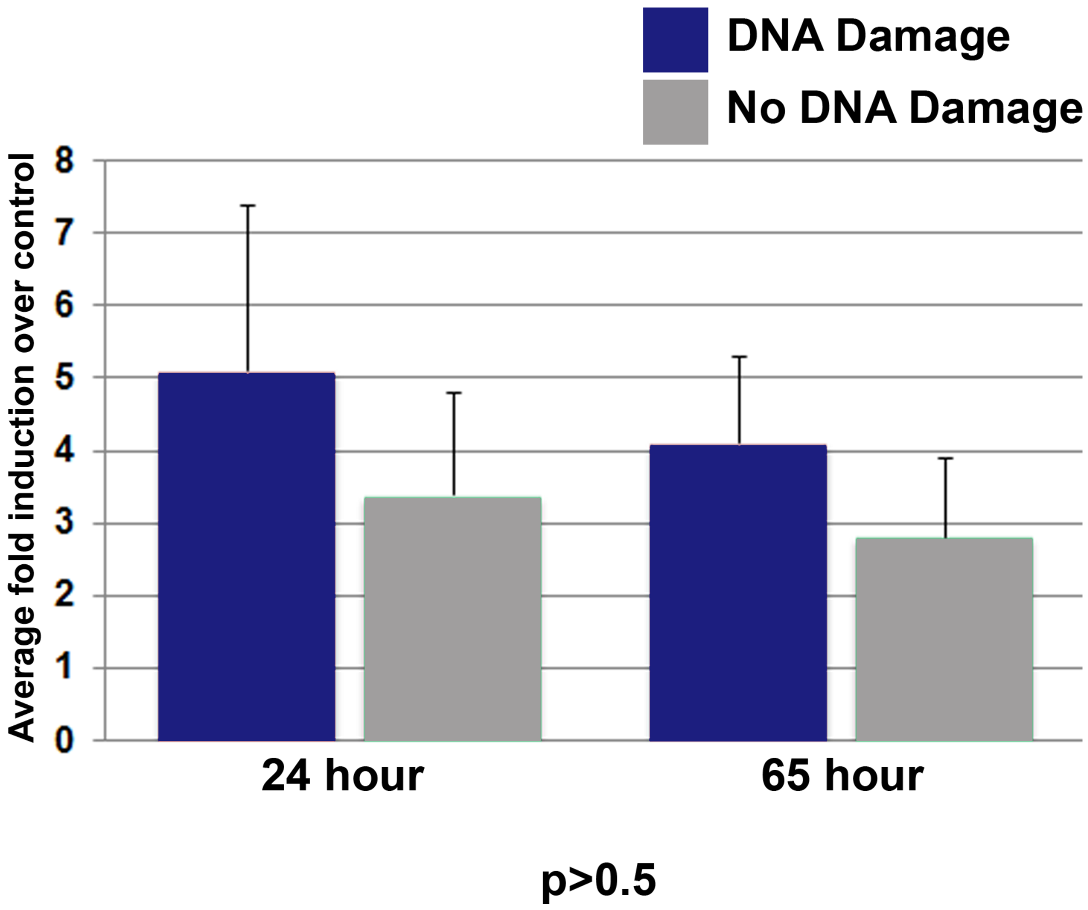

Supplemental Material, Figure S2. Genotoxicity of the screen hits.

Statistical analysis of the *C. elegans* screen comparing fold induction of the chemicals that cause hepatic DNA damage according to the ToxCast database (blue) against those that do not (grey). The two groups are not significantly different from each other at either time point.

Table S1. Aneugenicity of chemotherapeutic agents. Literature evidence used to determine the aneugenicity of the chemotherapeutic agents used in the small-scale screen.

CAS #	Chemical	Mechanism of action explaining its aneugenicity	Literature evidence supporting mechanism of action and aneugenicity
24386-93-4	5-iodo-tubercidin	N/A	N/A
153436-53-4	AG1478	N/A	N/A
315300	Allopurinol	N/A	N/A
179324697	Bortezomib	Proteasome inhibition	Bonvini et al. 2007; Strauss et al. 2007
50760	Dactinomycin	DNA binding/Transcription inhibition	Sobell 1985; Howlett et al. 2000
59052	Methotrexate	Dihydrofolate reductase inhibition	Rajagopalan et al. 2002; Finley 1975
31430-18-9	Nocodazole	Microtubule poison	De Brabander et al. 1977; Generoso et al. 1989
51183	Triethylenemelamine (TEM)	Alkylating agent, precise mode of action unknown	Luippold et al. 1978; Kamiguchi et al. 2002
154427	Thioguanine	DNA synthesis inhibition	Lepage 1960; Finley 1975
119413546	Topotecan HCl	Topoisomerase inhibition	Staker et al. 2002; Attia et al. 2009
122520-86-9	Tyr47	N/A	N/A
143679	Vinblastine sulfate	Microtubule poison	Yang et al. 2010; Russo and Pacchierotti 1988; Satya-Prakash et al. 1984
57-22-7	Vincristine	Microtubule poison	Novichkova et al. 2003; Martin et al. 1995; Subramanyam et al. 1984

Table S2. Screening results ranked by fold induction over control (24-hour exposure) and selected *in vivo* assays from ToxRefDB with lowest-observed-adverse-effect level (LOAEL) used to ascertain mammalian reprotoxicity.

CAS #	Chemical	Fold induction/DMSO in *C. elegans* Screen	MGR_Rat_Fertility	MGR_Rat_Implantations	MGR_Rat_LitterSize	MGR_Rat_Ovary	MGR_rat_ReproductiveOutcome	MGR_rat_ReproductivePerformance	MGR_Rat_ViabilityPND4
68694-11-1	Triflumizole	47.43434791	0	0	8.5	0	8.5	1.5	8.5
333-41-5	Diazinon	29.7344074	35.2	0	35.2	0	35.15	35.15	35.2
27314-13-2	Norflurazon	26.80981954	0	0	0	0	0	102.5	0
60168-88-9	Fenarimol	5.027203902	2.5	0	0	0	1.2	2.5	0
148-79-8	Thiabendazole	4.931905275	0	0	0	0	0	0	0
5598-13-0	Chlorpyrifos-methyl	4.890771735	0	0	0	0	0	0	0
298-00-0	Parathion-methyl	4.769592644	0	0	0	0	0	0	2
2310-17-0	Phosalone	4.516556087	0	0	29.4	0	29.4	0	29.4
115-32-2	Dicofol	4.161666966	0	0	0	2.4	23.2	0	11.2
51-03-6	Piperonyl butoxide	3.158182843	0	0	0	0	0	0	0
80-05-7	Bisphenol A	3.04040093	0	500	500	500	500	500	0
34256-82-1	Acetochlor	2.885046944	0	0	0	0	0	0	0
21564-17-0	TCMTB	2.783634106	0	0	0	0	0	0	0
40487-42-1	Pendimethalin	2.530258905	0	0	215	0	215	0	250
119446-68-3	Difenoconazol	2.395538652	0	0	0	0	0	0	0
22781-23-3	Bendiocarb	2.39407467	0	0	0	0	0	0	0
82097-50-5	Triasulfuron	2.252722533	0	0	0	0	0	0	0
87820-88-0	Tralkoxydim	2.206018524	0	0	0	0	0	0	0
12427-38-2	Maneb	2.006029049	0	0	0	25.1	0	106	0
50471-44-8	Vinclozolin	1.879949692	74.7	0	0	15.8	290	74.71	290
19666-30-9	Oxadiazon	1.796300837	0	0	0	0	0	0	0
63-25-2	Carbaryl	1.788232823	0	0	31.3	0	31.34	0	31.3
96489-71-3	Pyridaben	1.640636518	0	0	0	2.41	0	0	0
7696-12-0	Tetramethrin	1.602601904	0	0	0	0	0	0	0
23103-98-2	Pirimicarb	1.476808188	0	0	0	0	0	0	0
116714-46-6	Novaluron	1.455516443	0	0	895	0	895	0	0
1897-45-6	Chlorothalonil	1.430615893	0	0	0	0	0	0	0
199119-58-9	Trifloxysulfuron-sodium	1.362272788	0	0	0	0	0	0	0
23184-66-9	Butachlor	1.328392372	0	0	0	0	0	0	150
55406-53-6	3-Iodo-2-propynylbutylcarbamate	1.244443088	0	0	0	0	37.5	0	0

CAS #	Chemical	Fold induction/DMSO in *C. elegans* Screen	MGR_Rat_Fertility	MGR_Rat_Implantations	MGR_Rat_LitterSize	MGR_Rat_Ovary	MGR_rat_ReproductiveOutcome	MGR_rat_ReproductivePerformance	MGR_Rat_Viability PND4
54593-83-8	Chlorethoxyfos	1.181500188	0	0	0	0	0	0	0
127277-53-6	Prohexadione-calcium	1.17990055	0	0	0	0	0	0	0
34014-18-1	Tebuthiuron	1.112288164	0	0	0	0	0	0	0
53112-28-0	Pyrimethanil	1.054219846	0	0	0	0	0	0	0
07-01-18	Mancozeb	0.986173306	0	0	0	0	0	0	0
21087-64-9	Metribuzin	0.875461148	0	0	0	0	0	0	0
741-58-2	Bensulide	0.862246464	68.2	0	0	0	0	68.2	86.5
149979-41-9	Tepraloxydim	0.830711013	0	0	0	0	0	0	0
101200-48-0	Tribenuron-methyl	0.805891586	0	0	0	0	0	0	0
1918-02-1	Picloram	0.780617035	0	0	0	0	0	0	0
181274-15-7	Propoxycarbazone-sodium	0.780231293	0	0	0	0	0	0	0
23135-22-0	Oxamyl	0.61974946	0	0	0	0	0	0	11.6
58-89-9	Lindane	0.502707967	0	0	0	0	0	0	13.1
115-29-7	Endosulfan	0.453807115	0	0	0	0	0	0	0
99-30-9	Dichloran	0.437982988	0	0	0	102	0	0	0
126833-17-8	Fenhexamid	0.2784784	0	0	0	0	0	0	0
161050-58-4	Methoxyfenozide	0	0	0	0	0	0	0	0
122-14-5	Fenitrothion [a]	21.20687734	N/A	N/A	N/A	N/A	N/A	N/A	N/A
50-65-7	Niclosamide [a]	3.045456699	N/A	N/A	N/A	N/A	N/A	N/A	N/A
2971-36-0	HPTE [a]	1.812340306	N/A	N/A	N/A	N/A	N/A	N/A	N/A
110488-70-5	Dimethomorph [a]	1.740194007	N/A	N/A	N/A	N/A	N/A	N/A	N/A
120-32-1	Clorophene [a]	1.61369554	N/A	N/A	N/A	N/A	N/A	N/A	N/A
72-43-5	Methoxychlor [a]	1.151684234	N/A	N/A	N/A	N/A	N/A	N/A	N/A
67747-09-5	Prochloraz [a]	0.697528462	N/A	N/A	N/A	N/A	N/A	N/A	N/A

[a] Compound with no associated ToxRefDB data.

Table S3. Screening results ranked by fold induction over control (65-hour exposure) and selected *in vivo* assays from ToxRefDB with lowest-observed-adverse-effect level (LOAEL) used to ascertain mammalian reprotoxicity.

CAS #	Chemical	Fold induction/DMSO in *C. elegans* Screen	MGR_Rat_Fertility	MGR_Rat_Implantations	MGR_Rat_LitterSize	MGR_Rat_Ovary	MGR_rat_ReproductiveOutcome	MGR_rat_ReproductivePerformance	MGR_Rat_ViabilityPND4
333-41-5	Diazinon	27.52264231	35.2	0	35.2	0	35.15	35.15	35.2
68694-11-1	Triflumizole	12.14193182	0	0	8.5	0	8.5	1.5	8.5
27314-13-2	Norflurazon	11.1266272	0	0	0	0	0	102.5	0
298-00-0	Parathion-methyl	10.12097475	0	0	0	0	0	0	2
115-32-2	Dicofol	7.856347018	0	0	0	2.4	23.2	0	11.2
21564-17-0	TCMTB	6.545360335	0	0	0	0	0	0	0
148-79-8	Thiabendazole	6.315093921	0	0	0	0	0	0	0
12427-38-2	Maneb	4.860559196	0	0	0	25.1	0	106	0
22781-23-3	Bendiocarb	4.58531799	0	0	0	0	0	0	0
58-89-9	Lindane	4.166842845	0	0	0	0	0	0	13.1
96489-71-3	Pyridaben	3.738556866	0	0	0	2.41	0	0	0
50471-44-8	Vinclozolin	3.640963394	74.7	0	0	15.8	290	74.71	290
126833-17-8	Fenhexamid	2.528525438	0	0	0	0	0	0	0
07-01-18	Mancozeb	2.28223463	0	0	0	0	0	0	0
55406-53-6	3-Iodo-2-propynylbutylcarbamate	2.054130749	0	0	0	0	37.5	0	0
99-30-9	Dichloran	1.801286408	0	0	0	102	0	0	0
51-03-6	Piperonyl butoxide	1.708748513	0	0	0	0	0	0	0
80-05-7	Bisphenol A	1.69223028	0	500	500	500	500	500	0
34256-82-1	Acetochlor	1.691834611	0	0	0	0	0	0	0
1918-02-1	Picloram	1.633641901	0	0	0	0	0	0	0
21087-64-9	Metribuzin	1.618990138	0	0	0	0	0	0	0
7696-12-0	Tetramethrin	1.565990352	0	0	0	0	0	0	0
1897-45-6	Chlorothalonil	1.485886822	0	0	0	0	0	0	0
63-25-2	Carbaryl	1.445492186	0	0	31.3	0	31.34	0	31.3
5598-13-0	Chlorpyrifos-methyl	1.356563917	0	0	0	0	0	0	0
19666-30-9	Oxadiazon	1.151418533	0	0	0	0	0	0	0
82097-50-5	Triasulfuron	1.147528119	0	0	0	0	0	0	0
149979-41-9	Tepraloxydim	1.093244966	0	0	0	0	0	0	0
23184-66-9	Butachlor	1.042137896	0	0	0	0	0	0	150
741-58-2	Bensulide	0.987180898	68.2	0	0	0	0	68.2	86.5
23135-22-0	Oxamyl	0.940980345	0	0	0	0	0	0	11.6

CAS #	Chemical	Fold induction/DMSO in *C. elegans* Screen	MGR_Rat_Fertility	MGR_Rat_Implantations	MGR_Rat_LitterSize	MGR_Rat_Ovary	MGR_rat_ReproductiveOutcome	MGR_rat_ReproductivePerformance	MGR_Rat_ViabilityPND4
53112-28-0	Pyrimethanil	0.93334423	0	0	0	0	0	0	0
119446-68-3	Difenoconazol	0.899413506	0	0	0	0	0	0	0
116714-46-6	Novaluron	0.897406799	0	0	895	0	895	0	0
2310-17-0	Phosalone	0.894588344	0	0	29.4	0	29.4	0	29.4
60168-88-9	Fenarimol	0.855335862	2.5	0	0	0	1.2	2.5	0
161050-58-4	Methoxyfenozide	0.833130623	0	0	0	0	0	0	0
40487-42-1	Pendimethalin	0.827708683	0	0	215	0	215	0	250
181274-15-7	Propoxycarbazone-sodium	0.809788487	0	0	0	0	0	0	0
34014-18-1	Tebuthiuron	0.797573805	0	0	0	0	0	0	0
199119-58-9	Trifloxysulfuron-sodium	0.75711426	0	0	0	0	0	0	0
23103-98-2	Pirimicarb	0.709465026	0	0	0	0	0	0	0
127277-53-6	Prohexadione-calcium	0.623827781	0	0	0	0	0	0	0
87820-88-0	Tralkoxydim	0.620743192	0	0	0	0	0	0	0
101200-48-0	Tribenuron-methyl	0.27784166	0	0	0	0	0	0	0
115-29-7	Endosulfan	0.211630061	0	0	0	0	0	0	0
54593-83-8	Chlorethoxyfos	0.192832285	0	0	0	0	0	0	0
122-14-5	Fenitrothion [a]	25.17315023	N/A	N/A	N/A	N/A	N/A	N/A	N/A
50-65-7	Niclosamide [a]	3.408636186	N/A	N/A	N/A	N/A	N/A	N/A	N/A
110488-70-5	Dimethomorph [a]	3.00095051	N/A	N/A	N/A	N/A	N/A	N/A	N/A
72-43-5	Methoxychlor [a]	1.738985481	N/A	N/A	N/A	N/A	N/A	N/A	N/A
2971-36-0	HPTE [a]	1.276960831	N/A	N/A	N/A	N/A	N/A	N/A	N/A
120-32-1	Clorophene [a]	0.935294699	N/A	N/A	N/A	N/A	N/A	N/A	N/A
67747-09-5	Prochloraz [a]	0.691727971	N/A	N/A	N/A	N/A	N/A	N/A	N/A

[a] Compound with no associated ToxRefDB data.

Table S4. Germline defects quantification.

Quantification of the germline defects observed following exposure to 4 compounds: DMSO, Maneb, TCMTB and Dicofol.

Compounds	Normal (%)	Disorganized meiotic stages (%)	Germline nuclei loss/gaps (%)	Unevenly spaced nuclei (%)	Total number of germlines
DMSO	9 (90)	0 (0)	1 (10)	0 (0)	10
Maneb	4 (23.5)	5 (29.5)	6 (35)	2 (12)	17
TCMTB	7 (47)	5 (33)	3 (20)	0 (0)	15
Dicofol	9 (100)	0 (0)	0 (0)	0 (0)	9

```
###############################################################################
##Author: Nicole Kleinstreuer
##Date: August, 2012
##
## Calculate relative risk values for each chosen MGR_endpoint by iteratively
## varying cutoffs of fold change response (observed values) in C Elegans
## Two timepoints: 24 hr and 65 hr
##
## Questions to nicole.kleinstreuer@nih.gov
###############################################################################

rm(list=ls())

data.input.dir <- "C:/C elegans P allard/Celegans_data/"
data.file <- "RR_Data.csv"

celegans.data <- read.csv(file=paste(data.input.dir,data.file,sep=""),header=TRUE)

n<-length(celegans.data$Chemical)

celegans.stats.any<-matrix(data=NA, nrow = n-1, ncol = 13)
colnames(celegans.stats.any)<-
c("Cutoff","TP","TN","FP","FN","PPV","NPR","Sens","Spec","BA","RR","CI1","CI2")

cutoff <- celegans.data$Avg_24

celegans.data.sorted<-celegans.data[order(cutoff),]

for (c in 1:8){
        endpoint <- colnames(celegans.data.sorted)[c+4]
        endpoint.data <- celegans.data.sorted[,c+4]

        for (i in 1:n-1){
                celegans.stats.any[i,1] <- celegans.data.sorted$Avg_24[i]
                TP <- sum(endpoint.data[(i+1):n])
                celegans.stats.any[i,2] <- TP
                TN <- i-sum(endpoint.data[1:i])
                celegans.stats.any[i,3] <- TN
                FP <- (n-i)-TP
                celegans.stats.any[i,4] <- FP
                FN <- sum(endpoint.data[1:i])
                celegans.stats.any[i,5] <- FN
                PPV <- TP/(TP+FP)
                celegans.stats.any[i,6] <- PPV
                NPR <- FN/(TN+FN)
                celegans.stats.any[i,7] <- NPR
                Sens <- TP/(TP+FN)
                celegans.stats.any[i,8] <- Sens
                Spec <- TN/(FP+TN)
                celegans.stats.any[i,9] <- Spec
                BA <- (Sens + Spec)/2
                celegans.stats.any[i,10] <- BA
                RR <- PPV/NPR
                celegans.stats.any[i,11] <- RR
```

```
                s <- FP/(TP*(TP+FP)) + TN/(FN*(FN+TN))
                CI1 <- exp(log(RR)-1.96*s)
                celegans.stats.any[i,12] <- CI1
                CI2 <- exp(log(RR)+1.96*s)
                celegans.stats.any[i,13] <- CI2

        }

write.csv(celegans.stats.any,file=paste(data.input.dir,"Celegans_Analysis_24hr_",endpoint,
".csv",sep=""),row.names=FALSE)
}

cutoff <- celegans.data$Avg_65

celegans.data.sorted<-celegans.data[order(cutoff),]

for (c in 1:8){
        endpoint <- colnames(celegans.data.sorted)[c+4]
        endpoint.data <- celegans.data.sorted[,c+4]

        for (i in 1:n-1){
                celegans.stats.any[i,1] <- celegans.data.sorted$Avg_65[i]
                TP <- sum(endpoint.data[(i+1):n])
                celegans.stats.any[i,2] <- TP
                TN <- i-sum(endpoint.data[1:i])
                celegans.stats.any[i,3] <- TN
                FP <- (n-i)-TP
                celegans.stats.any[i,4] <- FP
                FN <- sum(endpoint.data[1:i])
                celegans.stats.any[i,5] <- FN
                PPV <- TP/(TP+FP)
                celegans.stats.any[i,6] <- PPV
                NPR <- FN/(TN+FN)
                celegans.stats.any[i,7] <- NPR
                Sens <- TP/(TP+FN)
                celegans.stats.any[i,8] <- Sens
                Spec <- TN/(FP+TN)
                celegans.stats.any[i,9] <- Spec
                BA <- (Sens + Spec)/2
                celegans.stats.any[i,10] <- BA
                RR <- PPV/NPR
                celegans.stats.any[i,11] <- RR
                s <- FP/(TP*(TP+FP)) + TN/(FN*(FN+TN))
                CI1 <- exp(log(RR)-1.96*s)
                celegans.stats.any[i,12] <- CI1
                CI2 <- exp(log(RR)+1.96*s)
                celegans.stats.any[i,13] <- CI2

        }

write.csv(celegans.stats.any,file=paste(data.input.dir,"Celegans_Analysis_65hr_",endpoint,
".csv",sep=""),row.names=FALSE)
}
```

References

Attia SM, Aleisa AM, Bakheet SA, Al-Yahya AA, Al-Rejaie SS, Ashour AE, Al-Shabanah OA. 2009. Molecular cytogenetic evaluation of the mechanism of micronuclei formation induced by camptothecin, topotecan, and irinotecan. Environ Mol Mutagen. Mar;50(2):145-51.

Bonvini P, Zorzi E, Basso G, Rosolen A.. 2007. Bortezomib-mediated 26S proteasome inhibition causes cell-cycle arrest and induces apoptosis in CD-30+ anaplastic large cell lymphoma. Leukemia. Apr;21(4):838-42. Epub 2007 Feb 1.

De Brabander M, De May J, Joniau M, Geuens G. 1977. Ultrastructural immunocytochemical distribution of tubulin in cultured cells treated with microtubule inhibitors. Cell Biol Int Rep. Mar;1(2):177-83.

Finley WH. 1975. Effect of drugs on chromosome structure. Am J Clin Nutr. May;28(5):521-9.

Generoso WM, Katoh M, Cain KT, Hughes LA, Foxworth LB, Mitchell TJ, Bishop JB. 1989. Chromosome malsegregation and embryonic lethality induced by treatment of normally ovulated mouse oocytes with nocodazole. Mutat Res. Feb;210(2):313-22.

Howlett NG, Schiestl RH. 2000. Simultaneous measurement of the frequencies of intrachromosomal recombination and chromosome gain using the yeast DEL assay. Mutat Res. Nov 6;454(1-2):53-62.

Kamiguchi Y, Tateno H. 2002. Radiation- and chemical-induced structural chromosome aberrations in human spermatozoa. Mutat Res. Jul 25;504(1-2):183-91.

Lepage GA. 1960. Incorporation of 6-thioguanine into nucleic acids. Cancer Res. Apr;20:403-8.

Luippold HE, Gooch PC, Brewen JG. 1978. The production of chromosome aberrations in various mammalian cells by triethylenemelamine. Genetics. Feb;88(2):317-26.

Martin RH, Rademaker AW, Leonard NJ. 1995. Analysis of chromosomal abnormalities in human sperm after chemotherapy by karyotyping and fluorescence in situ hybridization (FISH). Cancer Genet Cytogenet. Mar;80(1):29-32.

Novichkova EA, Onishchenko GE, Shtil AA. 2003. Microtubule depolymerization by vincristine causes cell death after transition from C mitosis to interphase. Dokl Biol Sci. Nov-Dec;393:575-8.

Rajagopalan PT, Zhang Z, McCourt L, Dwyer M, Benkovic SJ, Hammes GG. 2002. Interaction of dihydrofolate reductase with methotrexate: ensemble and single-molecule kinetics. Proc Natl Acad Sci U S A. Oct 15;99(21):13481-6.

Satya-Prakash KL, Hsu TC, Wheeler WJ. 1984. Metaphase arrest, anaphase recovery and aneuploidy induction in cultured Chinese hamster cells following exposure to mitotic arrestants. Anticancer Res. Nov-Dec;4(6):351-6.

Sobell HM. 1985. Actinomycin and DNA transcription. Proc Natl Acad Sci U S A. Aug;82(16):5328-31.

Staker BL, Hjerrild K, Feese MD, Behnke CA, Burgin AB Jr, Stewart L. 2002. The mechanism of topoisomerase I poisoning by a camptothecin analog. Proc Natl Acad Sci U S A. Nov 26;99(24):15387-92. Epub 2002 Nov 8.

Strauss SJ, Higginbottom K, Jüliger S, Maharaj L, Allen P, Schenkein D, Lister TA, Joel SP. 2007. The proteasome inhibitor bortezomib acts independently of p53 and induces cell death via apoptosis and mitotic catastrophe in B-cell lymphoma cell lines. Cancer Res. Mar 15;67(6):2783-90.

Subramanyam S, Laxminarayana D, Helen KD. 1984. Evaluation of genotoxic potential of vincristine from multiple parameters. Mutat Res. Oct;138(1):55-62.

Yang H, Ganguly A, Cabral F. 2012. Inhibition of cell migration and cell division correlates with distinct effects of microtubule inhibiting drugs. J Biol Chem. Oct 15;285(42):32242-50. doi: 10.1074/jbc.M110.160820. Epub 2010 Aug 9.
